# Fermented Durian Tempoyak as a Source of Probiotics for Colorectal Cancer Prevention through Gut Microbiome Modulation

**DOI:** 10.1007/s11894-026-01043-4

**Published:** 2026-05-07

**Authors:** Wing Soon Ng, Nancy Choon-Si Ng, Rebecca Shin-Yee Wong, Bey Hing Goh

**Affiliations:** 1 Department of Biomedical Sciences, Sir Jeffrey Cheah Sunway Medical School, Faculty of Medical and Life Sciences, Sunway University, Sunway City, Malaysia; 2 Department of Medical Education, Sir Jeffrey Cheah Sunway Medical School, Faculty of Medical and Life Sciences, Sunway University, Sunway City, Malaysia; 3 Faculty of Education and Liberal Arts, INTI International University, Nilai, Malaysia; 4 Faculty of Medicine, University of Cyberjaya, Cyberjaya, Malaysia; 5 Faculty of Health, Australian Research Centre in Complementary and Integrative Medicine, University of Technology Sydney, Ultimo, NSW Australia; 6 Sunway Biofunctional Molecules Discovery Centre, Faculty of Medical and Life Sciences, Sunway University, Sunway City, Malaysia; 7 Graduate Institute of Cancer Biology and Drug Discovery, College of Medical Science and Technology, Taipei Medical University, Taipei, Taiwan

**Keywords:** Colorectal cancer, Gut microbiome, Fermented foods, Probiotics, Tempoyak

## Abstract

**Purpose of Review:**

Colorectal cancer (CRC) remains a major global and Malaysian public health concern, with increasing recognition of gut dysbiosis as a contributor to colorectal tumorigenesis. This review examines fermented durian tempoyak as a culturally relevant, probiotic-rich traditional food with potential application in CRC prevention through gut microbiome modulation.

**Recent Findings:**

Dysbiosis may promote CRC through disruption of gut barrier integrity, chronic mucosal inflammation, immune dysregulation, reactive oxygen species (ROS)-mediated DNA damage, and altered microbial metabolism leading to carcinogenic metabolites such as secondary bile acids and hydrogen sulphide. Tempoyak commonly contains lactic acid bacteria, particularly *Lactiplantibacillus plantarum*, as well as *Limosilactobacillus fermentum* and *Levilactobacillus brevis*. Preclinical evidence suggests that related LAB strains can attenuate NF-κB, MAPK, STAT3, IL-17, and COX-2-associated inflammatory pathways, reduce immune-cell infiltration and oxidative stress, restore mucus and tight junction proteins, modulate bile acid metabolism, and reduce tumor burden in CRC or colitis-associated CRC models.

**Summary:**

Current evidence supports the mechanistic plausibility of tempoyak-associated LABs as microbiome-based agents for CRC chemoprevention. However, direct evidence using tempoyak-derived strains remains limited, and translation is constrained by strain-specific effects, microbial variability, sensory acceptability, safety and standardisation issues, and uncertain LAB viability after cooking. Future studies should prioritise strain characterisation, starter culture standardisation, probiotic stabilisation strategies, CRC-specific preclinical models, and well-designed human trials in high-risk populations.

## Introduction

Colorectal cancer (CRC) refers to any cancer that starts in the colon or rectum. In 2022, CRC was ranked by GLOBOCAN as the third most commonly diagnosed cancer and the second leading cause of cancer-related mortality worldwide [1]. In Malaysia, CRC has a poor prognosis in stages III and IV, with a 5-year survival rate of 18.4%, and is more common among the Chinese population (47.3%) and males (55.9%) [2]. These statistics warrant greater emphasis on early colorectal cancer prevention. Moreover, CRC has been associated with non-modifiable risk factors such as age and gender, as well as modifiable risk factors such as physical inactivity, unhealthy diet, smoking, and obesity [3]. There is also increasing evidence supporting the role of gut microbiota and its metabolites in the tumorigenesis of CRC [4–6].

A microbiota is a collection of microbes such as bacteria, yeasts, and viruses that are present in a specific environment of the host [7]. The human microbiota is further classified based on the site of colonisation, such as skin microbiota, eye microbiota, oral microbiota, vaginal microbiota, and gut microbiota [8]. In the past decade, the gut microbiota has received increasing attention from researchers worldwide [9]. The gut microbiota plays an important role in digestion, nutrient uptake, nutrient synthesis, immunomodulation, and can be affected by a variety of factors such as diet, intestinal pH, gut motility, host immune system, and more [10]. This highlights the importance of maintaining a healthy gut microbiota to prevent diseases.

**Fig. 1:**
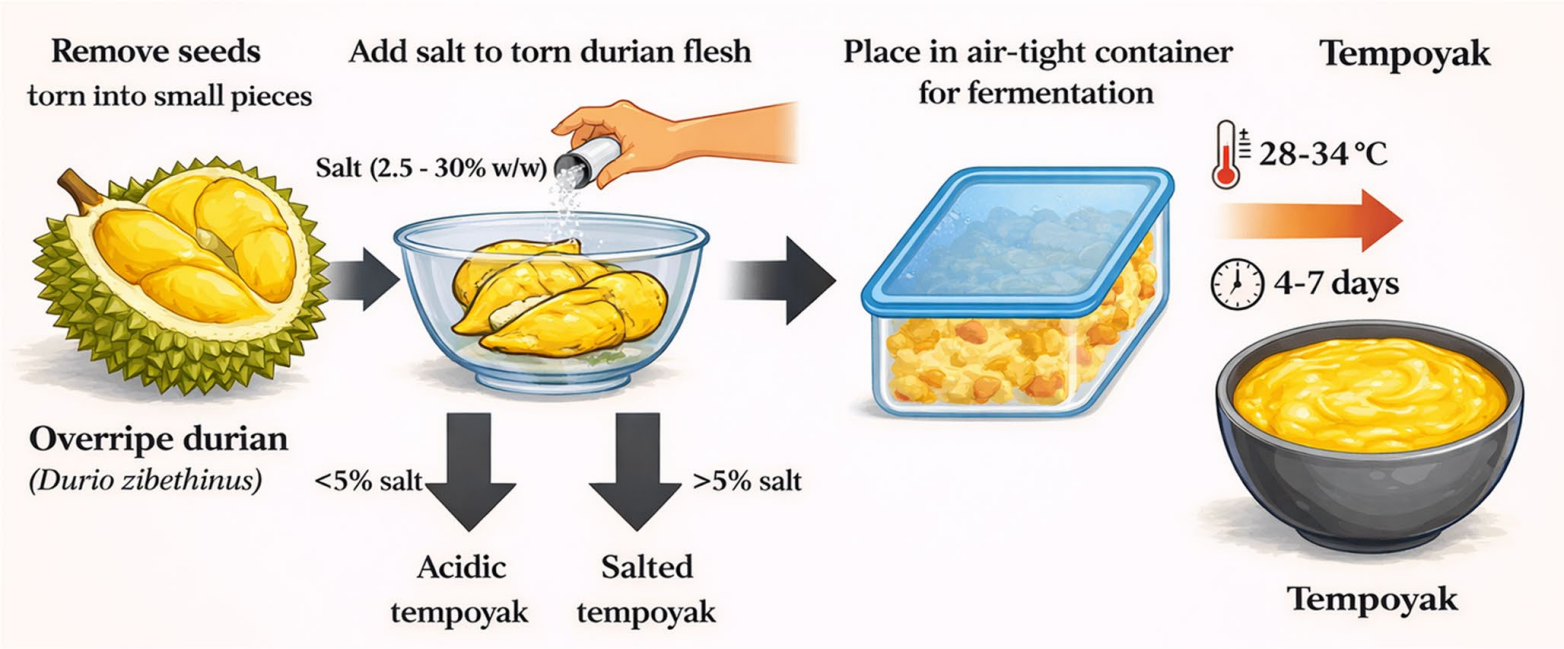
Traditional process of preparing fermented durian tempoyak through lactic acid fermentation. Overripe durian flesh is mixed with salt and fermented in an airtight container under anaerobic or semi-anaerobic conditions for several days, promoting the natural enrichment of lactic acid bacteria. This spontaneous fermentation process produces tempoyak, a traditional fermented food that serves as a potential source of probiotics relevant to gut microbiome modulation

Tempoyak, also known as “pekasam” or “durian asam” is a traditional Malay condiment that involves the making of durian paste through lactic acid fermentation [11]. The process of making tempoyak involves the fermentation of overripe durian flesh with salt in an air-tight container as shown in Fig. 1 [11, 12]. Tempoyak contains various strains of LAB, predominantly *Lactiplantibacillus plantarum* (*L. plantarum*; formerly *Lactobacillus plantarum*), that are promising probiotic candidates [11, 13, 14]. The characterisation of LABs from tempoyak raises important questions regarding their potential in preventing CRC via gut microbiota modulation and their broader public health implications. Therefore, this review examines key mechanisms in dysbiosis-associated CRC pathogenesis and evaluates existing preclinical evidence on the chemopreventive effects of selected LABs to support the exploration of tempoyak as a natural source of CRC-preventive probiotics.

## Methodology

This narrative review was informed by a structured literature search conducted using the PubMed and Scopus databases to identify relevant studies on gut microbiota, CRC pathogenesis, and probiotic interventions, particularly lactic acid bacteria (LAB) derived from fermented foods such as tempoyak. Articles published between 2015 and 2025 were prioritised, with earlier seminal studies included where necessary to provide foundational context. The search strategy incorporated combinations of keywords including “colorectal cancer,” “gut microbiota,” “dysbiosis,” “lactic acid bacteria,” “probiotics,” “fermented foods,” and “tempoyak.”

Studies were selected based on their relevance to the objectives of this review, particularly those examining mechanistic links between gut dysbiosis and CRC, as well as preclinical or clinical evidence evaluating the effects of probiotics or LAB on CRC-related pathways such as inflammation, gut barrier integrity, and microbial metabolite production. Articles not available in English, lacking mechanistic or experimental relevance to CRC, or focusing on unrelated disease contexts were excluded. The final selection of studies was guided by their scientific quality, relevance, and contribution to the overall narrative synthesis, rather than through a formal systematic review process.

### Gut Dysbiosis and Colorectal Cancer Pathophysiology

Gut dysbiosis may increase colorectal cancer risk through several interconnected mechanisms, beginning with disruption of gut barrier integrity, followed by chronic inflammation and altered microbial metabolism that promotes carcinogenic metabolite production. These mechanisms interact to create a pro-tumorigenic colonic environment. The relevant literature on dysbiosis-associated CRC pathogenesis is summarised in Table 1.

**Table 1:** Overview of dysbiosis-associated mechanisms implicated in colorectal cancer pathogenesis

Mechanism	Key biological processes	Key signalling pathways	CRC-relevant outcomes	Representative taxa	References
Chronic inflammation	• Pro-inflammatory cytokine overproduction • Macrophage activation • Accumulation of reactive oxygen and nitrogen species • Persistent immune activation	• NF-κB • COX-2 • MAPK • STAT3 • Th17-dependent signalling	• Enhanced cell proliferation • Angiogenesis • Inhibition of apoptosis • Oncogene activation • Tumour suppressor gene inactivation	• *Enterococcus faecalis* • Enterotoxigenic *Bacteroides fragilis* • *Fusobacterium nucleatum* • *Helicobacter pylori* (CagA-positive strains) • pks-positive *Escherichia coli* • *Streptococcus bovis*	Artemev et al. (25); Grellier et al. (18); Ionescu et al. (22)
Carcinogenic microbial metabolites	• Overproduction of hydrogen sulphide • Increased secondary bile acid formation • Bacterial genotoxin production (e.g. colibactin) • Dysregulation of TMAO and polyamine metabolism	• Ras/MAPK • Wnt/β-catenin • PI3K/AKT/mTOR • TGR5 • EGFR	• Direct DNA damage • Genomic instability • Tumour suppressor inhibition • Enhanced cancer cell stemness and invasiveness	• *Campylobacter jejuni* • *Clostridium perfringens* • *Clostridium ramosum* • Enterotoxigenic *Bacteroides fragilis* • *Fusobacterium nucleatum* • pks-positive *Escherichia coli* • Sulfidogenic bacteria (e.g. *Bilophila*, *Desulfovibrio*, *Desulfomicrobium*)	Artemev et al. (25); Liu et al. (24); Moon et al. (49)
Gut barrier dysfunction	• Increased intestinal permeability • Loss of tight junction integrity • Thinning of the mucus layer • Dysregulation of short-chain fatty acids	• β-catenin • STAT3	• Bacterial translocation • Sustained mucosal inflammation • Facilitation of tumour initiation and progression	• Enterotoxigenic *Bacteroides fragilis* • *Fusobacterium nucleatum* • pks-positive *Escherichia coli* • *Streptococcus gallolyticus*	Coleman et al. (16); Genua et al. (21)

#### Gut Barrier Dysfunction

Gut barrier dysfunction is a key early event linking gut dysbiosis to colorectal carcinogenesis. Importantly, it also serves as a central link integrating dysbiosis-associated inflammation and carcinogenic metabolite production. As part of the gut barrier, the mucus layer created by epithelial goblet cells is responsible for preventing gut bacteria from coming in direct contact with the colonocytes, and it is upregulated by gut commensals and short-chain fatty acids (SCFAs) [15]. Moreover, the mucus layer can facilitate microbe-mucus interactions where pathogens could potentially alter the host mucus to favour the growth of a pathogenic microflora [16]. Additionally, gastrointestinal (GI) diseases such as irritable bowel disease (IBD) and cancer have been associated with impaired intestinal mucus function [16]. Furthermore, Pothuraju et al. [17] demonstrated that mucin 4 (Muc4) knockout transgenic murine models had increased colonic polyps, colonic crypt hyperplasia, reduced colonic mucus layer, increased bacterial infiltration, decreased mucin 2 (Muc2) expression, and increased nuclear β-catenin accumulation, further supporting gut barrier dysfunction-associated CRC initiation. However, the relevant molecular mechanisms involving Muc4 remain to be elucidated. Furthermore, the aforementioned carcinogenic metabolites have also been implicated in gut barrier dysfunction [18, 19]. For example, a preclinical study done by Yang et al. [20] demonstrated that enterotoxigenic *Bacteroides fragilis* (ETBF) and its secreted genotoxin, *B. fragilis* toxin (BFT) increased colorectal tumor, induced intestinal mucosal barrier damage, downregulated Muc2, and upregulated phosphorylated STAT3 (p-STAT3) proteins, supporting the role of microbial genotoxins in gut barrier dysfunction and CRC pathogenesis. Besides that, Genua et al. [21] also stated that gut barrier dysfunction can exacerbate gut inflammation via increased translocation of pro-inflammatory microbial metabolites, increased exposure of bacteria to colonocytes, and hyperactivation of the immune system. Importantly, disruption of the mucus layer and epithelial tight junctions increases contact between luminal microbes, microbial products, and host immune cells, thereby facilitating persistent inflammatory signalling that further drives colorectal tumorigenesis.

#### Chronic Inflammation

Following impairment of gut barrier integrity, increased microbial translocation and epithelial exposure to pathogen-associated molecular patterns can promote persistent mucosal immune activation and chronic inflammation, which are major contributors to colorectal tumorigenesis. Ageing and lifestyle factors such as Western diet, sedentary lifestyle, and smoking can foster a pro-inflammatory gut microbiome profile [22]. For example, Bolte et al. [23] demonstrated through human faecal microbiome profiling the association between fast food consumption and increased abundance of *Parabacteroides johnsonii*, *Lachnospiraceae bacterium*, *Clostridium bolteae*, *Blautia*, and *Ruminococcus*. Furthermore, they found that fast food diet is associated with increased intestinal inflammatory markers, suggesting a link between diet-induced gut dysbiosis and gut inflammation. Interestingly, *P. johnsonii* had been shown in murine models to hinder CRC onset and progression via gut microbiota modulation [24]. This contrasting result establishes the need to further study the role of *P. johnsonii* in human subjects. Additionally, altered host environment may lead to the active and passive selection of pathogenic microbes which further encourage inflammation [25]. The invasion of tumorigenic pathogens and tissue inflammation will then lead to the production of reactive oxygen species (ROS) and reactive nitrogen species (RNS), as well as the activation of various signalling pathways involved in tumorigenesis.

The most studied signalling pathway where gut dysbiosis can contribute to chronic inflammation is the nuclear factor κB (NF-κB) pathway [26]. Pathogen-associated molecular patterns (PAMPs) such as lipopolysaccharide (LPS) can activate the canonical NF-κB pathway, which leads to the expression of pro-inflammatory cytokines such as tumor necrosis factor (TNF), interleukin 1 (IL-1), interleukin 6 (IL-6) and various chemokines [27]. Indeed, mice with dextran sulphate sodium (DSS)-induced gut dysbiosis and ulcerative colitis (UC) have increased serum NF-κB p65 and IL-6 levels, which are attenuated by faecal microbiota transplant [28]. Moreover, the activation of NF-κB by toll-like receptor 4 (TLR4) can lead to the overexpression of cyclooxygenase 2 (COX-2), a major inflammatory effector [22]. In an n-6 high-fat diet-induced gut dysbiosis murine model, the expression of colonic COX-2 is elevated by three-fold and accompanied by hyperplastic lesions, suggesting the tumorigenic role of COX-2 overproduction due to gut dysbiosis [29]. The production of COX-2 is also associated with gut pathogens such as *Enterococcus faecalis* (Efa) and *Streptococcus bovis* (*S. bovis*) [25]. Additionally, other pathways involved in chronic inflammation include mitogen-activated protein kinase (MAPK), signal transducer and activator of transcription 3 (STAT3), and T helper 17 (Th17)-dependent signalling pathway [18, 22, 25].

#### Role of Probiotics in Immunomodulation and ROS-Induced Carcinogenesis

From an immunological standpoint, probiotics may mitigate CRC risk via modulation of host immune responses, resulting in the attenuation of chronic inflammation and oxidative stress involved in ROS-mediated carcinogenesis.

Dysbiosis-associated colorectal tumorigenesis is driven by immune dysregulation, where immune cells such as Th17 cells, neutrophils, and M1 macrophages contribute to a tumorigenic microenvironment via chronic inflammation and oxidative stress. Gut dysbiosis has been correlated with Th17 dysregulation in IBD [30]. Furthermore, Cui et al. (2024), found that IL-17 A-positive Th17 cells were more abundant in dysplastic tissues of UC patients [31]. Therefore, dysbiosis-induced Th17 expansion may play a role in inflammation-associated colorectal carcinogenesis. In addition to their direct pro-inflammatory role in CRC pathogenesis [22, 25], Th17 cells may play an indirect role in carcinogenesis via the constant recruitment of neutrophils and macrophages to tumor sites [32]. To support this, Zhang et al. (2023), conducted a secondary data analysis and discovered elevated infiltration of neutrophils, M0 macrophage, and M1 macrophage in UC samples compared to healthy controls [33]. Consistent infiltration of tumor-associated neutrophils (TAN) during chronic inflammation leads to a tumorigenic microenvironment due to the release of ROS, RNS, and myeloperoxidase (MPO) [34]. Similarly, M1 tumor-associated macrophages (TAMs) may generate high levels of pro-inflammatory cytokines and ROS into the tumor microenvironment (TME) [35]. Despite its tumoricidal role, prolonged elevated levels of ROS can eventually lead to DNA damage-induced cellular transformation [36, 37]. Indeed, Todorović et al. (2024) found that colonic adenocarcinoma had double the frequency of 8-hydroxy-2′-deoxyguanosine (8-OHdG) compared to healthy tissues [38]. Moreover, high levels of ROS can also lead to abnormal activation of several pro-tumorigenic signaling pathways such as the PI3K/AKT pathway and the JAK/STAT pathway, leading to cell proliferation and apoptosis inhibition [39]. In short, immune hyperactivation caused by dysbiosis-induced chronic inflammation drives oxidative stress which results in oxidative DNA damage, aberrant activation of signaling pathways, and subsequent cellular transformation towards a dysplastic phenotype.

Probiotics such as LABs may attenuate dysbiosis-associated colorectal carcinogenesis through modulating host immune response and reducing oxidative stress implicated in ROS-mediated malignant transformation. For example, both *L. plantarum* YYC-3 and *L. brevis* CLB3 have been shown to reduce immune cells infiltration and IL-17 levels [40, 41], a Th17-associated cytokine involved in neutrophil recruitment and M1 macrophage polarization [42, 43]. Moreover, *L. plantarum* HNU082 has been shown to reduce colonic neutrophil infiltration and cytokine markers associated with M1 macrophages such as TNF-α, IL-1β, and IL-6 [44, 45]. Similarly, both L. fermentum GR3 and *L. fermentum* E7 reduced M1-associated cytokine markers while reducing lipid peroxidation markers and increasing systemic antioxidant levels [46, 47]. As ROS-mediated DNA damage and cellular transformation play key roles in colorectal carcinogenesis, these results support the mechanistic potential of tempoyak-associated LABs in mitigating CRC risk by regulating immune responses and attenuating oxidative stress.

#### Carcinogenic Metabolites

Alongside barrier disruption and chronic inflammation, gut dysbiosis also reshapes microbial metabolism, leading to accumulation of carcinogenic metabolites that further promote colorectal tumorigenesis. Gut dysbiosis plays a role in CRC pathogenesis via dysregulation of microbial metabolism, leading to an increased production of carcinogenic metabolites such as secondary bile acids (SBAs), hydrogen sulphide (H2S), trimethylamine N-oxide (TMAO), and bacterial genotoxins. Recently, there was an increased interest in studying the role of SBAs in carcinogenesis. SBAs such as deoxycholic acid (DCA), lithocholic acid (LCA), and ursodeoxycholic acid (UDCA) are produced by the intestinal microflora via 7α-dehydroxylation of free primary bile acids (PBAs) in the intestines [48]. While UDCA is suggested to be gut protective, high colonic DCA and LCA levels are implicated in colorectal carcinogenesis via multiple mechanisms such as genomic instability, direct DNA and mitochondrial damage, inhibition of apoptosis, and activation of epidermal growth factor receptor (EGFR) signalling and Wnt signalling pathway [48]. However, the current research on SBAs is relatively immature and preclinical, requiring further studies to establish a strong mechanistic link with CRC in human subjects.

Besides that, dysbiosis-associated H2S production had also been implicated in CRC aetiology [19, 25]. H2S is mainly produced by gut sulfidogenic bacteria such as *Bilophila*, *Desulfovibrio*, *Desulfomicrobium*, and *Fusobacterium* via metabolism of organic and inorganic sulphur compounds [49]. Supporting this, Grion et al. [50] showed that the abundance of *Desulfovibrio* and *Bilophila* is elevated in faecal samples of CRC patients compared to healthy controls. Moreover, a prospective cohort study by Wang et al. [51] demonstrated an increased risk of CRC in participants following a sulphur microbial diet. Overall, these results suggest an association between dysbiosis-associated alterations in sulphur metabolism and CRC aetiology. H2S is believed to play a role in carcinogenesis via causing DNA damage and genomic instability, promoting tumor cell growth and proliferation, as well as inducing angiogenesis and vasorelaxation [25, 49]. However, it was noted that the harmful effects of endogenous H2S are associated at millimole concentrations, whereas micromole concentrations of H2S exhibit beneficial effects such as vasorelaxation, endoplasmic reticulum (ER) stress reduction, and apoptosis prevention [49]. This further supports the carcinogenic potential of gut dysbiosis via altered production of metabolites. Other carcinogenic metabolites such as TMAO and bacterial genotoxins (e.g. colibactin) are implicated in increased risk of CRC but are relatively underexplored in human subjects compared to SBAs and H2S, establishing a need for further research.

Collectively, these findings support a sequential and interconnected model in which gut dysbiosis first compromises barrier integrity, thereby promoting chronic inflammation and altered microbial metabolite production, all of which contribute to CRC pathogenesis.

Given that gut barrier disruption, chronic inflammation, and carcinogenic microbial metabolites are central to dysbiosis-associated CRC pathogenesis, probiotic strategies that can modulate these processes warrant closer attention. In this context, LABs isolated from tempoyak are of particular interest because preclinical evidence suggests that related strains may act across several of these mechanistic pathways.

### Chemopreventive Potential of Probiotics Isolated from Tempoyak

The most common LAB isolated from tempoyak regardless of origin is *L. plantarum* [11, 12]. Other notable species include *Pediococcus acidilactici* (*P. acidilactici*), *Leuconostoc mesenteroides* (*L. mesenteroides*), *Levilactobacillus brevis* (*L. brevis*), and *Limosilactobacillus fermentum* (*L. fermentum*) [11, 12]. This is further supported by Basuki et al. [52] and Murwani et al. [14] where they isolated various strains of *L. plantarum* from Indonesian tempoyak. In this review, the anti-CRC potential of *L. plantarum*, *L. fermentum*, and *L. brevis* will be discussed.

#### *L. plantarum*

*L. plantarum* is the most promising probiotic candidate isolated from tempoyak, showcasing viability under simulated gastric, intestinal, and bile salt conditions [13]. Beyond that, *L. plantarum* strains isolated from non-tempoyak sources have been shown in preclinical CRC models to modulate gut microbiota composition, attenuate gut inflammation, repair intestinal barrier, and reduce tumor burden (Table 2).

**Table 2:** Preclinical evidence on the chemopreventive potential of *Lactiplantibacillus plantarum* in colorectal cancer

Organism	Strain (source)	Study design	Key findings	References
*Lactiplantibacillus plantarum*	12 (turbot intestine)	In vivo	• Reduced number of colonic tumours • Decreased pro-inflammatory cytokines (IL-8, IL-1β, TNF-α) • Restored IL-10 levels • Increased goblet cell number and restored crypt architecture • Upregulated Claudin-1 expression • Downregulated NF-κB p65, phosphorylated p65 and p38 MAPK • Increased IκB-α • Increased pro-apoptotic Bax expression • Altered gut microbiota composition with reduced *Proteobacteria*, *Desulfovibrionaceae* and *Helicobacteraceae* and increased *Lactobacillaceae*, *Bifidobacteriaceae* and *Muribaculaceae*	Ma et al. (53)
	YYC-3 (fermented rose juice)	In vivo	• Prevented tumour formation • Reduced leukocyte infiltration • Decreased IL-6, IL-17 F and IL-22 levels • Suppressed phosphorylation of IκB-α and NF-κB p65 • Inhibited nuclear β-catenin accumulation • Restored gut microbiota composition towards a healthy phenotype	Yue et al. (41)

*Bax*, Bcl-2–associated X protein; *IL*, interleukin; *MAPK*, mitogen-activated protein kinase; *NF-κB*, nuclear factor kappa B; *TNF-α*, tumour necrosis factor alpha

Notably, Ma et al. [53] found that *L. plantarum-*12 isolated from turbot intestine is able to reduce levels of pro-inflammatory cytokines and inhibit NF-κB and MAPK signalling pathway, which was previously mentioned to be implicated in dysbiosis-associated CRC onset. Likewise, Yue et al. [41] demonstrated that oral administration of *L. plantarum* YYC-3 isolated from fermented rose juice ameliorated signs of inflammation and suppressed pro-inflammatory signalling pathways in CRC murine models, which resulted in reduce tumor formation. Moreover, Fareez et al. [54] demonstrated that *L. plantarum* LAB12 strain greatly reduced COX-2 expression in tumor tissues of orthotopic CRC mice. Together, these findings suggest that *L. plantarum* may exert chemopreventive effects against CRC via modulation of gut inflammation.

Although studies on *L. plantarum* and its potential in reducing carcinogenic microbial metabolites are lacking, *L. plantarum*-12 has been shown to reduce the abundance of *Desulfovibrionaceae* family, which includes major sulfidogenic genera such as *Bilophila* or *Desulfovibrio*, suggesting the potential role of *L. plantarum* probiotics in lowering dysbiosis-associated overproduction of genotoxic H2S via sulphur metabolism modulation. However, further in vivo studies examining the H2S level after L. plantarum supplementation are required to support this proposed mechanism. Even though Padro et al. [55] showed that *L. plantarum* strains with high bile salt hydrolase (BSH) activity can modulate bile acid metabolism in healthy overweight individuals via reduction of circulating conjugated BAs, no differences in faecal SBAs were noted after probiotic supplementation. This raises questions on the effectiveness of *L. plantarum* in reducing carcinogenic SBAs such as LCA and DCA in the colonic environment. Therefore, future studies are required to elucidate the role of *L. plantarum* on bile acid metabolism regulation specifically in the context of CRC.

Additionally, *L. plantarum* has been shown to restore gut barrier integrity. For example, *L. plantarum-*12 was shown to restore colonic goblet cells and colonic crypt structures, as well as upregulate tight junction protein Claudin-1 expression [53]. Moreover, Wu et al. [45] revealed that *L. plantarum* HNU082 recovered tight junction protein zonula occludens-1 (ZO-1) and Muc2 expression in the colon of DSS-induced UC mice model. Although the study was done in a UC model, UC was associated with an increased CRC risk via shared mechanisms including gut dysbiosis, chronic inflammation, carcinogenic microbial metabolites, and gut barrier dysfunction [56]. Consequently, these findings provide indirect yet biologically relevant evidence supporting the gut barrier-protective role of *L. plantarum* probiotics in mitigating CRC risks.

#### *L. fermentum*

Another probiotic candidate shown preclinically to inhibit CRC pathogenesis is *L. fermentum*. Similar to *L. plantarum*, *L. fermentum* has been shown to ameliorate inflammatory markers, modulate bile acid metabolism, and restore gut barrier integrity (Table 3), the key carcinogenic pathways associated with CRC.

**Table 3:** Preclinical evidence on the chemopreventive potential of *Limosilactobacillus fermentum* in colorectal cancer

Organism	Strain (source)	Study design	Key findings	References
*Limosilactobacillus fermentum*	ZS40(traditionally fermented yoghurt)	In vitro and in vivo	In vitro: • Maintained viability under simulated gastric and bile salt conditions In vivo: • Reduced colon shortening and tumour burden • Decreased serum inflammatory markers (IL-1β, IL-8, TNF-α, MIP-1β, VCAM-1)• Reduced inflammatory infiltration • Reduced inflammatory infiltration • Downregulated colonic NF-κB-related genes (p65, IKKβ, TRAF-6, COX-2) • Upregulated anti-apoptotic and regulatory genes (IκB-α, TRAF-1/2, Bcl-2, Bcl-xL) • Reduced expression of tumour markers CD34 and CD117	Liu et al. (57)
	GR-3(traditional fermented food Jiangshui)	In vitro and in vivo	In vitro: • Strong antioxidant and free radical scavenging activity • Induced apoptosis and atrophy in CRC cell lines • Upregulated p53 and Bax and downregulated Bcl-2 expression • Produced bioactive indole metabolites In vivo: • Reduced colon shortening, dysplasia, tumour number and inflammatory infiltration • Restored goblet cells and mucosal structure· Enhanced barrier integrity via upregulation of Muc2, TFF3, ZO-1 and occluding • Suppressed NF-κB, β-catenin, TLR4 and pro-inflammatory cytokines • Modulated gut microbiota composition· Increased antioxidant capacity, glutathione and superoxide dismutase • Reduced lipid peroxidation, LPS translocation, secondary bile acids (LCA, DCA) and serum permeability markers	Zhou et al. (46)

*Bax*, Bcl-2–associated X protein; *Bcl-2*, B-cell lymphoma 2; *CA*, cholic acid; *COX-2*, cyclooxygenase-2; *CRC*, colorectal cancer; *DCA*, deoxycholic acid; *IL*, interleukin; *IKK*, IκB kinase; *LCA*, lithocholic acid; *LPS*, lipopolysaccharide; *NF-κB*, nuclear factor kappa B; *TFF3*, trefoil factor 3; *TLR4*, toll-like receptor 4; *TNF-α*, tumour necrosis factor alpha; *VCAM-1*, vascular cell adhesion molecule 1; *ZO-1*, zonula occludens-1

A comprehensive preclinical study by Zhou et al. [46] demonstrated the capabilities of *L. fermentum* GR-3 isolated from a Chinese fermented food “Jiangshui” in attenuating dysbiosis-associated colitis by reducing colonic inflammatory infiltration, reducing pro-inflammatory cytokine and COX-2 levels, and downregulating the NF-κB signalling pathway in AOM/DSS-induced CRC mouse. This resulted in decreased colonic abnormalities and tumor burden, supporting the protective effects of *L. fermentum* on colitis-associated CRC tumorigenesis. Likewise, Liu et al. [57] showed that *L. fermentum* ZS40 isolated from yogurt reduced systemic pro-inflammatory cytokine and COX-2 levels, leading to decreased inflammatory infiltration and tumor burden in the colon of AOM/DSS-induced CRC mice. Therefore, these findings in similar CRC murine models support the anti-inflammatory and antitumor effects of *L. fermentum* supplementation.

Moreover, *L. fermentum* has showed potential in regulating carcinogenic microbial metabolite production via gut microbiome modulation. For instance, *L. fermentum* GR-3 supplementation resulted in decreased faecal LCA and DCA levels [46], the two major SBAs implicated in CRC carcinogenesis. In addition, GR-3 exhibited strong antioxidant activity and free radical scavenging capacity in vitro and in vivo. Hence, these findings suggest a mechanistic potential of *L. fermentum* in reducing CRC risk via regulation of SBA metabolism and oxidative stress. Nonetheless, further studies in examining sulphur metabolism and clinical trials with large sample size are needed to validate the CRC-preventive potential of *L. fermentum* isolated from tempoyak.

*L. fermentum* also showed impressive gut barrier protective properties through the restoration of gut barrier integrity and production of barrier-protective microbial metabolites. Notably, Zhou et al. [46] reported that GR-3 supplementation significantly upregulated expression of mucin-associated proteins Muc2 and trefoil factor-3 (TFF3), as well as tight junction proteins ZO-1 and occludin in the colon of CRC mice. This resulted in reduced systemic infiltration of fluorescein isothiocyanate (FITC)-dextran and LPS levels, suggesting amelioration of colitis-induced gut permeability. Likewise, Zhang et al. [47] reported that *L. fermentum* E7 increased MUC2, ZO-1, occludin, and claudin-1 expression in the colon of DSS-induced UC murine models, supporting its gut barrier–protective role.

#### *L. brevis*

In addition to *L. fermentum*, *Levilactobacillus brevis* (previously known as *Lactobacillus brevis*) is another potential probiotic isolated from tempoyak shown to retain viability in growth tolerance assays using simulated gastrointestinal and bile salt conditions [13]. However, studies on the anti-CRC effects of *L. brevis* remain scarce, with existing preclinical evidence largely restricted to the anti-inflammatory effects of *L. brevis* supplementation in CRC cell lines and murine models (Table 4).

**Table 4:** Preclinical evidence on the chemopreventive potential of *Levilactobacillus brevis* in colorectal cancer

Organism	Strain (source)	Study design	Key findings	References
*Levilactobacillus brevis*	CLB3 (fermented pickles)	In vitro and in vivo	In vitro: • High acid tolerance • Low bile salt resistance In vivo: • Reduced colon shortening, tumour size and severity • Decreased immune cell infiltration • Reduced IL-6 and IL-17 A levels • Downregulated Runx1, mTOR, SLC7A5, AKT and phosphorylated AKT • Reduced STAT3 and phosphorylated STAT3 expression • Decreased caspase-3 activation	Qian et al. (40)
	SBL8803 (Sapporo Breweries Ltd.)	In vitro and in vivo	In vitro: • Selectively inhibited colorectal cancer cell growth and induced apoptosis without affecting normal epithelial cells • Increased ERK phosphorylation In vivo: • Suppressed tumour growth in a mouse xenograft model	Sakatani et al. (58)

*AKT*, protein kinase B; *ERK*, extracellular signal-regulated kinase; *IL*, interleukin; *mTOR*, mechanistic target of rapamycin; *STAT3*, signal transducer and activator of transcription 3

In particular, Qian et al. [40] demonstrated that *L. brevis* CLB3 isolated from fermented pickles significantly reduced tumor growth and severity, attenuate inflammatory response, and inhibit IL-6/protein kinase B (AKT)/p-STAT3 signalling pathway in azoxymethane (AOM) /DSS-induced CRC murine models. Moreover, Sakatani et al. [58] further supported the antitumor effects of the postbiotic polyphosphate derived from *L. brevis* in CRC cell lines and mice xenograft model. Intriguingly, paraprobiotic *L. brevis* WB2357 isolated from kimchi has been shown to attenuate TNF-α/interferon-γ (IFN-γ)-induced pro-inflammatory cytokine production possibly via modulation of NF-κB/p38 MAPK and STAT3 signalling pathways in HaCaT cells [59]. In relevance to inflammation-associated CRC pathogenesis, these findings suggest that L. brevis may exhibit anti-inflammatory properties through its metabolites and bacterial cell components.

Currently, there is limited evidence on the effects of *L. brevis* supplementation on regulating carcinogenic microbial metabolites productions in relevant CRC models. However, Zhou et al. [60] demonstrated the potential of *L. brevis* D17 in regulating bile acid metabolism as a result of gut microbiota modulation in obese mice. While the study was done in high-fat diet (HFD) obese mice model, HFD and obesity are both implicated in increased CRC risk via dysregulation of bile acids and gut dysbiosis [48]. Thus, these findings suggest mechanistic plausibility to support future studies in examining the SBA modulatory effects of *L. brevis* in CRC murine models.

*L. brevis* supplementation has also been shown preclinically to be gut barrier protective, although studies done on CRC models are very limited. For instance, Shin et al. [61] demonstrated that *L. brevis* Bmb6 isolated from kimchi reduced DSS-induced goblet cell damage, restored mucus integrity, and upregulated colonic tight junction protein ZO-1 expression in DSS-induced colitis murine model. However, Čuljak et al. [62] reported variable gut barrier modulatory effects of different *L. brevis* strains in TNF-α and LPS-challenged CRC cell lines, establishing a need to validate the gut barrier protective effects using tempoyak-derived *L. brevis* strains. Nonetheless, these preclinical findings suggest a biological plausibility of *L. brevis* supplementation in restoring inflammation-associated gut barrier dysfunction, a key mechanism implicated in CRC pathogenesis.

Collectively, available evidence suggests that tempoyak-associated LABs may influence CRC-related pathways through three recurring mechanistic domains: attenuation of inflammatory signalling, modulation of carcinogenic microbial metabolites, and restoration of gut barrier integrity.

### Public Health Implications

Although current evidence remains largely preclinical, the cultural embeddedness and accessibility of tempoyak raise the possibility that its probiotic potential could be considered not only at the mechanistic level, but also within broader dietary prevention and public health frameworks.

Tempoyak represents a culturally relevant fermented food with potential public health impact as a microbiome-based dietary intervention for CRC prevention in Malaysia, owing to its affordability, simple production method, cultural significance, and nutritional value. Tempoyak’s affordability and ease of production stem from its two widely accessible ingredients in Malaysia: durian and salt. As one of the main producers of durian, Malaysia is capable of meeting both local and international demands [63]. Furthermore, tempoyak can be easily prepared at home with minimal steps (Fig. 1). Additionally, tempoyak is culturally embedded within the Malay ethnic group, which comprises 58.1% of Malaysia’s population. Collectively, the cost-effectiveness, accessibility, and cultural relevance of tempoyak position it as a realistic candidate for population-level dietary interventions aimed at preventing CRC, the second most common cancer in Malaysia [2].

The intended public health outcomes of implementing tempoyak as a CRC-preventive functional food are a reduction in CRC incidence and improvement in overall nutritional status at a population level. In a meta-analysis done by Liang et al. [64], cheese intake was associated with decreased CRC risk, while yogurt intake was associated with decreased risk of rectal cancer but not colon cancer. Similarly, a meta-analysis of randomised controlled trials (RCTs) by Kan et al. [65] revealed that probiotic supplementation was associated with reduced colorectal adenoma incidence but not CRC incidence. Critically, the authors noted limitations such as small sample size, short intervention duration, and insufficient follow-up periods. Therefore, these findings suggested a role of fermented foods and probiotics in preventing CRC carcinogenesis at the early stage, supporting the exploration of tempoyak as a CRC-preventive functional food. Beyond probiotics, tempoyak’s nutritional value is also attributed to naturally occurring bioactive compounds in ripe durian pulp, including polyphenols, carotenoids, vitamins, and minerals [66], supporting its role as a dietary component for improving overall nutritional status.

Therefore, tempoyak as an ethnically relevant traditional food with potential anti-CRC properties may be considered for inclusion in national dietary guidelines or health promotion campaigns. For example, it could be included in pre-existing frameworks such as the Malaysian Healthy Plates to encourage regular dietary inclusion, which is important for sustained probiotic effects. Tempoyak may also be explored for large-scale industrial production, although challenges in standardisation, safety assessment, and quality control of probiotics must be addressed. Moreover, future efforts should focus on product innovation [67], such as the development of novel recipes or snack formats to improve acceptance and long-term compliance, which are key contributors to the success of population-level dietary interventions aimed at CRC prevention.

### Challenges and Future Perspectives

**Fig. 2:**
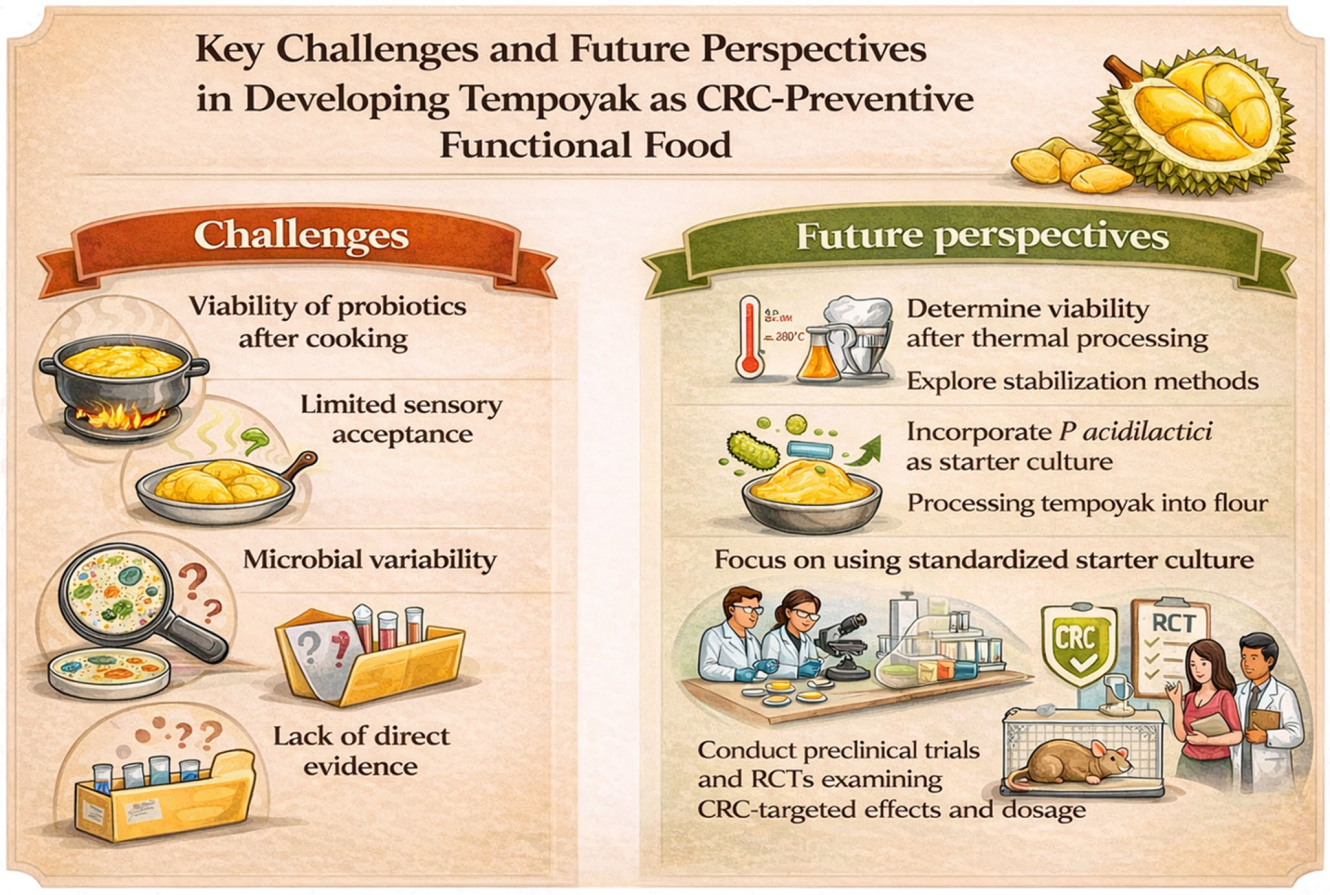
Key challenges and future perspectives in developing tempoyak as a microbiome-based functional food for colorectal cancer prevention. Major limitations include reduced probiotic viability following thermal processing, limited sensory acceptance due to strong aroma and acidity, microbial variability associated with spontaneous fermentation, and the lack of strain-specific preclinical and clinical evidence. Future strategies focus on improving probiotic stability through processing technologies, enhancing sensory acceptability, standardising fermentation using starter cultures, and generating mechanistic and clinical evidence to support translational application

Despite the mechanistic plausibility and public health appeal of tempoyak as a microbiome-based CRC-preventive food, several challenges continue to limit its translational potential. There are several challenges limiting the translatability of tempoyak as a source of CRC-preventive probiotics. The key challenges and future perspectives are summarised in Fig. 2. One of the main hurdles is the viability and stability of probiotics prior to consumption [68]. As a condiment, tempoyak is rarely consumed directly and is often added into cooked meals such as tempoyak curry or tempoyak fried rice in Malaysia [12], raising concerns regarding the effects of high temperatures on LAB viability during cooking. Generally, most LAB strains are unable to tolerate temperatures above 50 °C, with only a limited number of LAB strains being able to survive as high as 65 °C, while cooking temperatures above 100 °C are lethal to LABs [69]. This is an important consideration in the context of CRC prevention as live probiotics must reach the gut in order to colonise and exert its antitumor benefits. Further studies are required to determine the viability of LABs isolated from tempoyak after thermal processing and explore stabilisation methods such as spray drying or microencapsulation to increase viability of LABs in tempoyak upon consumption.

Another challenge in implementing tempoyak as a CRC-preventive functional food is limited sensory acceptance. Ripe durians are associated with a strong pungent sulphurous aroma [70] that may not be accepted by many. Furthermore, tempoyak is characterised by a pronounced sour taste due to lactic acid produced during fermentation, and its intensity is dependent on the amount of salt added before fermentation [12]. Hence, the combination of the natural sulphurous aroma of durian and the acidic flavour profile of tempoyak may restrict its acceptance among wider populations, which can potentially limit the frequency of its consumption and reduce its efficacy as a probiotic-rich food. Interestingly, Marwati and Apriadi [71] noted that using *P. acidilactici* as fermentation starter can reduce sulphur-containing compounds, which in turn could possibly minimise the inherent sulphurous aroma of durian. Moreover, Anggadhania et al. [12] suggested processing tempoyak into flour, which had been shown to reduce its sourness and extend shelf life. However, the impact of such processing on probiotic viability and stability remains to be studied in the future.

In addition, microbial variability also presents as a major obstacle in developing tempoyak as a standardised CRC-preventive fermented food. The microbial profile of tempoyak varies based on durian cultivar, geographic location, seasonality, and developmental stage of the durian [11]. Moreover, many homemade preparations prefer spontaneous fermentation as the main production method, which has been associated with increased risk of contamination by non-LAB species [11], raising concerns on the product safety and quality, as well as batch-to-batch variability and consistency. For this reason, future studies should focus on standardisation strategies such as using starter cultures (e.g. *P. acidilactici*) to minimise contamination risks and reduce microbial variability.

Despite the growing number of preclinical studies elucidating the potential antitumor effects of LABs isolated from fermented foods, there is a significant lack of preclinical and clinical studies done on the LAB strains isolated from tempoyak in the context of CRC. This limits the translatability of current evidence on LABs as certain effects of probiotics can be strain-specific. For example, *L. plantarum* S2T10D is a butyrate-producing strain while *L. plantarum* O2T60C is a non-producing strain [72]. Consequently, future studies are needed to examine the CRC-targeted effects and optimal dosage of tempoyak-derived probiotics in CRC cell lines and murine models. Additionally, RCTs involving high-risk populations for CRC would be required to evaluate its safety, efficacy, and clinical relevance of tempoyak as a microbiome-based, CRC-preventive functional food.

## Conclusion

Tempoyak is a traditional Malay fermented condiment and a natural source of LABs such as *L. plantarum*, *L. fermentum*, and *L. brevis* with emerging preclinical evidence supporting their mechanistic potential in mitigating CRC pathogenesis. These probiotics may exert their chemopreventive effects by targeting the four major dysbiosis-associated mechanisms: gut barrier dysfunction, chronic inflammation, immunomodulation and ROS-induced carcinogenesis, and carcinogenic microbial metabolite production. As a simple and affordable ethnic food, tempoyak is a potential candidate for population-level dietary interventions aimed at reducing CRC risk and improving nutritional status in Malaysia. However, challenges including reduced probiotic viability after cooking, limited sensory acceptance, microbial variability, and lack of direct preclinical and clinical evidence currently hinder its real-word application. Therefore, future studies should be focused on improving probiotic stability, enhancing sensory acceptance, standardising microbial composition, and providing causative preclinical and clinical evidence to support the translational potential of tempoyak as a microbiome-based, CRC-preventive functional food.

## Key References

- Tang B, Li S, Li X, He J, Zhou A, Wu L, et al. Cholecystectomy-related gut microbiota dysbiosis exacerbates colorectal tumorigenesis. Nat Commun. 2025;16:7638.
  - ○ Provides strong mechanistic evidence linking gut microbiome disruption to accelerated colorectal tumorigenesis, reinforcing dysbiosis as a causal driver rather than a bystander in CRC development.
- Tito RY, Verbandt S, Aguirre Vazquez M, Lahti L, Verspecht C, Lloréns-Rico V, et al. Microbiome confounders and quantitative profiling challenge predicted microbial targets in colorectal cancer development. Nat Med. 2024;30:1339–48.
  - ○ Highlights methodological limitations and confounding factors in CRC microbiome research, underscoring the need for careful interpretation of probiotic and microbial association studies.
- Van Hul M, Cani PD, Petitfils C, De Vos WM, Tilg H, El-Omar EM. What defines a healthy gut microbiome? Gut. 2024;73:1893–908.
  - ○ Offers a contemporary conceptual framework for defining a “healthy” gut microbiome, providing context for evaluating fermented foods such as tempoyak as microbiome-modulating dietary interventions.
- Anggadhania L, Setiarto RHB, Yusuf D, Anshory L, Royyani MF. Exploring tempoyak, fermented durian paste, a traditional Indonesian indigenous fermented food: typical of Malay tribe. J Ethn Food. 2023;10:42.
  - ○ Provides an up-to-date overview of tempoyak fermentation practices, microbial composition, and cultural relevance, supporting its selection as a probiotic-rich functional food candidate.
- Zhou T, Wu J, Khan A, Hu T, Wang Y, Salama E-S, et al. A probiotic Limosilactobacillus fermentum GR-3 mitigates colitis-associated tumorigenesis in mice via modulating gut microbiome. npj Sci Food. 2024;8:61.
  - ○ Demonstrates direct suppression of colitis-associated colorectal tumorigenesis through probiotic-mediated modulation of inflammation, bile acid metabolism, and gut barrier integrity.

## Data Availability

Data sharing is not applicable to this article as no new data were created or analysed in this study.
